# FDTD Simulation: Simultaneous Measurement of the Refractive Index and the Pressure Using Microdisk Resonator with Two Whispering-Gallery Modes

**DOI:** 10.3390/s20143955

**Published:** 2020-07-16

**Authors:** Ping Zhang, Dongyue He, Chen Zhang, Zhiruo Yan

**Affiliations:** School of Electrical and Information Engineering and Key Laboratory of Advanced Ceramics and Machining Technology of Ministry of Education, Tianjin University, Tianjin 300072, China; dongyue_he@tju.edu.cn (D.H.); 2019234325@tju.edu.cn (C.Z.); yanzr97@tju.edu.cn (Z.Y.)

**Keywords:** microdisk resonator, whispering-gallery mode, refractive index, pressure, finite-difference time-domain method

## Abstract

In this paper, an approach to measure both the refractive index (RI) and the pressure simultaneously using two Whispering-Gallery Modes (WGMs) in a microdisk resonator is theoretically proposed. Due to the difference in the energy distribution of the first and second order WGMs, the sensitivity of two modes toward the variation of RI and pressure applied to the device show differences. An RI sensitivity of 29.07 nm/RIU and pressure sensitivity of 0.576 pm/kPa for WGM (1,36), and an RI sensitivity of 38.68 nm/RIU and a pressure sensitivity of 0.589 pm/kPa for WGM (2,28) are obtained through the 3D finite-difference time-domain (3D-FDTD) simulation. Dual parametric sensing can be achieved by solving the second-order inverse sensitivity matrix. Therefore, strain–optical coupling behavior is effectively eliminated. The dual sensing scheme we proposed provides a novel approach to overcome the difficulty of multi-sensing applications based on the flexible photonic device.

## 1. Introduction

Recently, the demand for detection and analysis of different biochemical substances has been increasing dramatically in the fields of disease diagnosis, drug development, and environmental protection. Therefore, the study of biochemical sensors has become extremely important. Through the past few years, biochemical sensors using whispering-gallery mode (WGM) optical resonators have attracted widespread attention [1,2,3,4,5] due to the unique optical properties, fast response time, small size, and label-free-based sensor can provide detection down to the single-molecule level [6,7,8,9,10]. Theoretical research and numerical works have been widely studied in the field of nanoparticle sensing utilizing the WGM resonator [11,12,13,14,15], and various WGM optical resonators utilizing different structures, such as microsphere [16], microring [17], microdisk [18,19], micro-racetrack [20], microtoroid [21], microtube [22] and other shapes have been proposed and realized with outstanding performance for biological and chemical sensing. Among them, microdisk resonators have a unique position for their low cost, easy fabrication on-chip, and outstanding performance, such as their high quality factor, sensitivity and low detection limit.

At present, most conventional optical resonators are manufactured on rigid substrates such as semiconductors and glass which lack mechanical flexibility. Nevertheless, the mechanical stiffness of the substrates is incompatible with soft biological tissues [23], thus limiting application in the form of a flexible system, for example, the conformal sensor integration on human skin. The emerging flexible integrated photonic devices, which use flexible materials as substrates or fabricated with flexible materials such as polymers [24,25], help to solve the difficulty to some extent. Yu Chen et al. demonstrated that flexible photonic devices have been manufactured onto flexible substrates without compromising their function and performance [26]. Moreover, when combining the mechanical flexibility and excellent optical performance, the flexible optical micro-resonators have great potential for applications in optical ultrasonic sensor [27,28], optical accelerometer [29,30], optical strain sensor [31,32] and optical temperature sensor [33,34]. However, the sensing performance of flexible photonic resonators will be seriously damaged in the biochemical sensing application due to the non-biological impact on the device when experiencing mechanical deformation. The strain distribution in the device induced by large mechanical deformation will causes changes in the resonator dimension and the effective RI due to the strain–optical coupling effect [35], which subsequently leads to a shift of the resonant peak shift. Therefore, it is vital to eliminate the impact of strain–optical coupling behavior to realize the flexible biochemical sensor based on the WGM resonator. In this regard, Lan Li et al. proposed a multi-neutral-axis theory to calculate the strain distribution in a flexible integrated photonic device [35]. The strain exerted on the devices can be significantly reduced when placing them at the neutral plane location in advance. However, if the resonator is encapsulated in a thick cladding layer, the range of evanescent waves is difficult to extend the surface of the device to interact with the surrounding environment, which is an indispensable sensing condition. The working principle of most WGM resonator biosensors is the evanescent wave detection mechanism. Therefore, the sensitivity of the device to the pressure is a disadvantage for flexible biosensor, e.g., the refractive index sensors. Thus, distinguishing the shifts of resonance wavelength caused by the pressure and the RI variations during the measurement process is of great significance for flexible optical biosensors.

In order to eliminate the undesired effects such as temperature change, a lot of effort has been spent on developing dual parameter sensors. A microring resonator with dual-polarization [36] and a microdisk resonator with two WGMs [37] have been demonstrated for the simultaneous detection of RI and temperature. Analogously, it is feasible for a microdisk resonator with two WGMs to achieve dual parameter sensing of the RI and the pressure simultaneously. Moreover, we have successfully proposed a SOMRR structure for the simultaneous measurement of RI and pressure utilizing the mode splitting in our previous work [38]. However, the Q factor of the SOMRR is ~1000, much lower than state-of-the-art microcavity sensors due to the scattering loss at the opening. The microdisk resonator we propose here can achieve the same dual sensing and possess a high *Q* factor (~5990) and thus an enhanced RI detection limit down to ~8.96 × 10^−5^ RIU. Meanwhile, this proposed microdisk with a small radius (2 μm) decreases the size of the device which is beneficial for integration and miniaturization, and the wider FSR (~60nm) helps to enlarge the detection range.

In this paper, we propose an optical microdisk resonator on a flexible plastic substrate (SU-8) with two WGMs to achieve simultaneous detection of the surrounding RI and the pressure applied to the resonator. Since two WGMs possess different energy distribution, the responses of the two modes to RI and pressure change is different, thus meeting the requirement of the dual parameter measurement. Accurate numerical results have been achieved through 3D FDTD simulation using a commercialized software FDTD solutions to obtain transmission responses and two mode sensitivities of RI and pressure. All the mechanical simulations for estimating the pressure sensing are investigated through FEM simulation by a commercialized software COMSOL. When a sensitivity matrix is defined, we can distinguish the shifts of resonance wavelength corresponding to the RI and pressure changes. Hence, this sensing scheme effectively eliminates the effect of strain–optical coupling and offers a new approach to overcome the difficulty of inaccurate biosensing detection results utilizing the flexible optical resonators.

## 2. Theoretical Model and Working Principle

**Figure 1:**
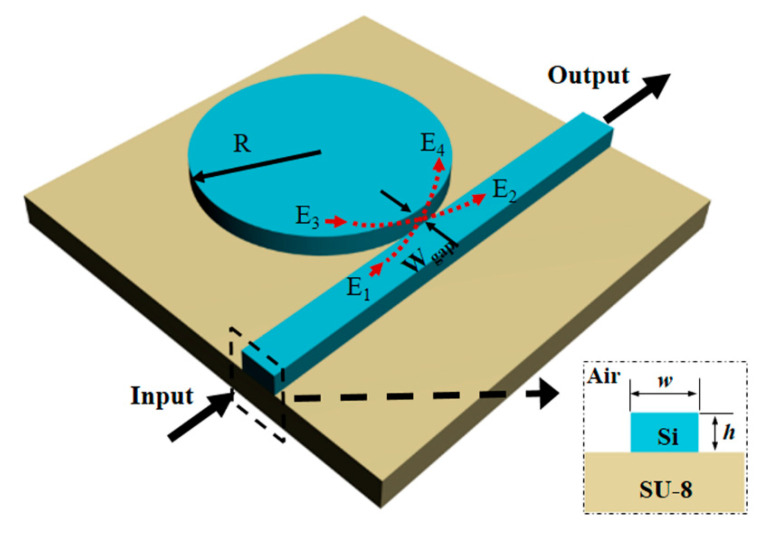
The diagram of the microdisk structure and the section dimension of the bus waveguide.

The schematic structure of the microdisk resonator (MDR) is shown in Figure 1. The radius of the disk is set: R = 2 μm. The width and the height of the bus waveguide are denoted as *w* and *h*, respectively. The coupling distance between the bus waveguide and the MDR is labeled as *Wgap*. The material of the microdisk cavity is silicon with an RI of 3.47 [39]. The RI of the surrounding environment is set as 1, representing an air cladding. Here, an epoxy (SU-8) with the RI of 1.56 [40] is selected as the plastic material of the flexible substrate, which is obviously different from the previously reported microdisk structure.

The light source with a particular wavelength band is injected at the input port of the bus waveguide. The total internal reflection of light rays along a curved boundary of the resonator causes the light ray to propagate in the form of WGMs. The resonance conditions can be expressed as

(1) $$2\pi Rn_{eff}=m\lambda$$

where *n_eff_* is the effective RI of the resonator cavity, and *m* is a positive integer, which represents the angular momentum. Therefore, the resonant wavelength will shift with the change of *n_eff_*, which is the crucial sensing principle for WGM micro-resonators in biosensing applications.

As shown in Figure 1, The relationship of electric field amplitude of each part can be described as follows by using the transmission matrix method:

(2) $$\left\{ \begin{array}{l} E_{2}=\tau E_{1}+ikE_{3}\\ E_{4}=ikE_{1}+\tau E_{3} \end{array}\right.$$

where *E_j_* (*j* = 1, 2, 3, 4) denotes the electric field amplitude of each part which are labeled in red arrows in Figure 1, *τ* and *k* are the amplitude self-coupling and cross-coupling coefficients between the bus waveguide and MDR, respectively. Usually in a lossless state, *τ*^2^ + *k*^2^ = 1. *E*_4_ can be described as

(3) $$E_{3}=\alpha e^{i\varphi }E_{4}$$

where *α* and *φ* are the intensity attenuation coefficient and the round-trip phase shift. From the Equations (2) and (3), the expression of the transmission spectrum of MDR can be expressed as

(4) $$T={\left|\frac{E_{2}}{E_{1}}\right|}^{2}=\frac{\alpha^{2}+\tau^{2}-2\alpha \tau \cos\varphi }{1+\alpha^{2}\tau^{2}-2\alpha \tau \cos\varphi }$$

**Figure 2:**
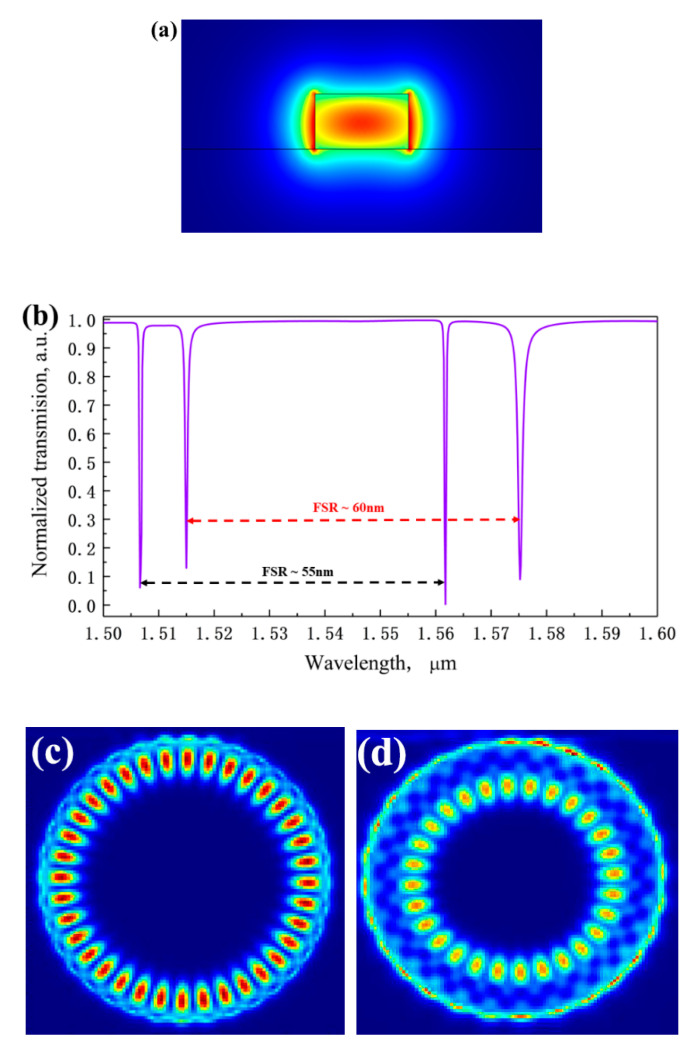
(**a**) The input TE mode’s energy distribution (**b**) The transmission spectrum of MDR with a particular wavelength band from 1.5 to 1.6 μm. The electric field distribution of the first order WGM (**c**) and the second order WGM (**d**).

In our work, all of the transmission spectra are obtained through the 3D FDTD simulation with software FDTD solutions. It is noted that the PML boundary condition has to be added at the outer boundaries during the simulation. The PML layers are set to be 8 in the x, y and z axes. Then, we optimize the parameters in the micro-cavity to get a good resonance state of the resonator. The disk radius is chosen as *R* = 2 μm. Both the microdisk resonator and bus waveguide have the same thickness and they are deposited on a 2-μm-thick substrate. The appropriate values of *w* and *h* for bus waveguide are designed to 390 and 230 nm, which ensures a single mode of transverse electric (TE) mode propagation in the structure. By setting the proper coupling distance *W_gap_*, 3D numerical simulations are performed and the mode profile of fundamental TE mode is in Figure 2a. The mesh accuracy of gridding is set to be 2, and the wavelength range of input source are from 1.5 to 1.6 μm. The high RI contrast between the cladding and waveguide core facilitates the confined light propagation in the waveguide. In Figure 2b, two resonance peaks appear in the normalized transmission spectrum corresponding to two WGMs. These two WGMs are the first order radial mode and the second order radial mode supported in our proposed MDR with a radius of 2 μm. Given their different free spectral ranges (FSR) and linewidths, the two groups of WGMs can be clearly distinguished. The FSR of two WGMs are ~55 and ~60 nm, respectively. Here, WGM (*v*, *m*) is defined to determine the characteristics of WGMs, where *v* is the radial mode order and *m* is the angular mode order. Hence, the two WGMs can be represented as WGM (1,36) with a resonance wavelength of ~1561 nm and WGM (2,28) with a resonance wavelength of ~1575 nm, and the mode profiles are shown in Figure 2c,d. We can clearly find that the electric field intensity distributions for the first two orders are quite different.

**Figure 3:**
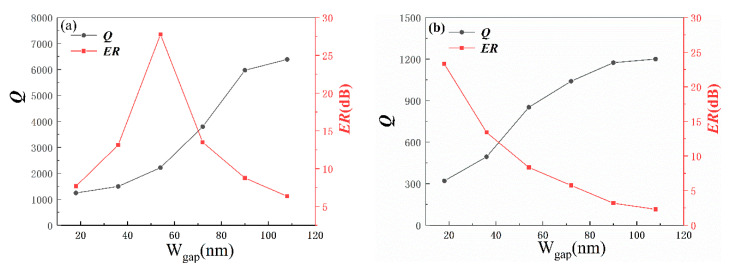
Q factor and ER of (**a**) WGM (1,36) and (**b**) WGM (2,28) as a function of *W_gap_*.

Then, the parameter of *W_gap_* is swept from 30 to 180 nm in a step of 30 to obtain the appropriate optical performance of MDR. The relationships between *Q*, Extinction Ratio *(ER)* and *W_gap_* are depicted in Figure 3.

To evaluate the performance of the MDR, including the sensitivity and the detection limit, the quality factor (*Q*) needs to be investigated emphatically. The quality factor (*Q*) is an important figure of merit used for all resonators and its physical meaning represents the number of round trips before the photon’s energy has decayed to 37% (1/e). *Q* is defined as the ratio of the resonance wavelength to the corresponding full width at half maximum of the resonant wavelength and can be described as *Q* = *λ*_0_/*FWHM*. The higher the *Q* value, the longer the photon circulation in the cavity. The strong photon storage also means an enhanced interaction between light and matter, which is beneficial for high-sensitivity sensing applications, and higher quality factors reduce the spectral noise of the sensor and improve the sensor’s detection limit. Another parameter, *ER*, can be defined as *ER* = 10*lg*(*P_max_*/*P_min_*), where *P_max_* and *P_min_* denote the maximum and minimum power at the output of the waveguide. High ER is more conducive to distinguish resonance peaks, thus reduces the influence of noise and help to minimize crosstalk. For WGM (1,36) in Figure 3a, *Q* monotonically increases with the increase of *W_gap_*, while ER first increases to the extreme, and then decreases as *W_gap_* increases. For WGM (2,28) in Figure 3b, the Q factor monotonically increases and ER monotonically decreases with the increase of *W_gap_*. Obviously, there is a trade-off between these two parameters. Due to higher *Q* is more important for sensing performance, the *W_gap_* is chosen to be 90 nm.

**Figure 4:**
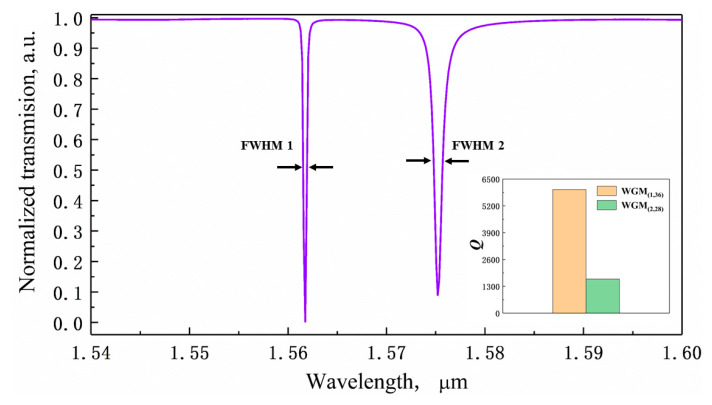
The transmission spectrum of MDR near the 1.55 μm wavelength band using FDTD simulation. The inset shows the comparison of the Q factor of two WGMs.

Under these optimized parameters, one complete period transmission spectrum of the MDR around the wavelength of 1.55 μm with two sets of resonances is shown in Figure 4 and *Q* factors of two WGMs are compared in the insert. The *Q* factor of the first order WGM (1,36) achieves ~5990, nearly four times that of second order WGM (2,28) (~1200).

## 3. Sensing Performance and Analysis

For biochemical sensors based on the optical WGM resonator, two sensing mechanisms are widely employed: surface and bulk (or homogeneous) sensing [41]. The former can achieve the specific detection of biochemical matter by binding of the preimmobilized functional sites on the surface of the microcavity and specific sensing molecules. The latter enables the concentration detection of biochemical molecules solution. Bulk sensing is mainly considered in the paper and it is also referred to as RI sensing. When the bulk RI of the entire cladding is altered, the effective RI of the resonator changes subsequently and the resonance wavelength occurs shifts according to Equation (1).

Sensitivity (*S*) and detection limit (*DL*) are the important parameters describing the performance of biosensors. Here, the RI (bulk) sensitivity (*S_RI_*) is described as the amount of wavelength shift per refractive index unit (RIU):

(5) $$S_{RI}=\frac{\delta \lambda }{\delta n}$$

Similarly, pressure sensitivity (*S_P_*) can be expressed as:

(6) $$S_{P}=\frac{\delta \lambda }{\delta P}$$

where *δλ* denotes the resonance wavelength shift induced by the RI or the pressure change, *δn* and *δP* are RI and pressure change, respectively. The *DL* can be derived by considering the noise in the transduction signal *δ*, i.e., the minimum resolvable signal: *DL = δ/S*. Therefore, the detection limit is dependent not only on the device, but also on the noise of the system. The transduction signal *σ* can be represented by the smallest detectable wavelength shift *δλ_min_*, which can be expressed by *δλ_min_* = *Fλ*/*Q*, where *F* is typically 1/50-1/100 [42]. Thus, *DL* can be expressed as

(7) $$DL=\frac{F\lambda }{QS}$$

A sensitivity matrix *M_RI,P_* is defined to estimate the dual parametric sensing performance as follows:

(8) $$M_{RI,\;P}=\left[\begin{array}{cc} S_{RI,\;WGM(1,36)} & S_{P,\;WGM(1,36)}\\ S_{RI,\;WGM(2,28)} & S_{P,\;WGM(2,28)} \end{array}\right]$$

where the subscript WGM (1,36) and WGM (2,28) identify the sensitivity for the corresponding first and the second order WGM. Therefore, the *δλ_WGM_*_(1,36)_ and *δλ_WGM_*_(1,28)_ induced by RI and pressure changes can be given by

(9) $$\left[\begin{array}{c} \delta \lambda_{WGM(1,36)}\\ \delta \lambda_{\;WGM(2,28)} \end{array}\right]=M_{RI,\;P}\times \left[\begin{array}{c} \delta n\\ \delta P \end{array}\right]$$

Each Sensitivity can be determined by monitoring the resonance wavelength shift of the WGM of the first two orders from the 3D FDTD simulation results.

### 3.1. The External RI Response of the MDR

**Figure 5:**
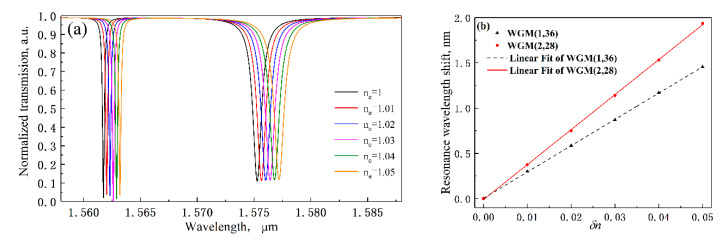
(**a**) The transmission spectra of the MDR with different RI of the surrounding environment. (**b**) Resonance wavelength shifts of the MDR versus the *δn*.

In order to measure the bulk sensitivity of the MDR sensor, the RI of the cladding is slightly changed from 1.01 to 1.05 in a step of 0.01. The corresponding simulated transmission spectrum is shown in Figure 5a. It is observed that the resonance bands of two WGMs occur red-shift with the increasing of surrounding RI. Then, the wavelength shift versus the relative change of RI is given in Figure 5b. One can find that the shift of the WGM (2,28) is larger than that of WGM (1,36). According to the fitting lines, we obtain the two RI sensitivities *S_RI,WGM_*_(1,36)_ =29.07nm/RIU and *S_RI,WGM_*_(2,28)_ = 38.68nm/RIU. Based on Equation (7), when *F* is chosen with 1/100, the corresponding RI detection limit are estimated to be approximately 8.96 × 10^−5^ RIU and 3.39 × 10^−4^ RIU.

**Figure 6:**
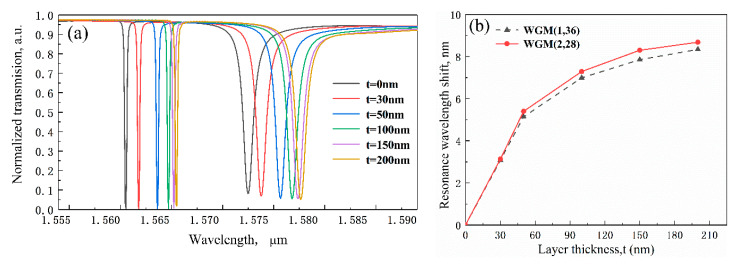
(**a**) The transmission spectra of the MDR with different adsorbed analyte layer thickness. (**b**) Resonance wavelength shifts of the MDR versus the layer thickness.

Then, to prove the surface sensing capacity of the MDR, a thin layer of biomolecule is attached on the surface of microdisk resonator to represent the processing of binding between target molecules and preimmobilized functional sites in the simulation. Here, we select protein with an RI of 1.48 [43] as the target biomolecule. Inspired by the research method on the biosensing characteristics of microring resonator in the literature [44], we change the thickness of adsorbed analyte layer to stand for different protein solution concentrations, and the sensing responses with the layer thickness of 0, 30, 50, 100, and 200 nm are, respectively, evaluated in Figure 6a. The resonance wavelength shifts versus the different layer thickness are shown in Figure 6b. Obviously, the resonance bands of two WGMs also occur in the red shift with the increasing of bio-layer thickness. From the detailed data in Figure 6b, resonance wavelength shift is no longer linear with bio-layer thickness. The slope of the curve is defined as surface sensitivity and can be expressed as *S* = *δλ*/*δt*, where *δt* is the change of layer thickness. Therefore, it is clear to see that the surface sensitivity increases linearly for small values of the layer thickness but exhibit a tendency for saturation as *t* increases beyond 50 nm. In other words, the MDR has a surface detection limit due to the limited surface area of the device and functional biorecepter. The change of surface sensitivity can be also explained as follows: Since the intensity of evanescent field decays exponentially outside the disk resonator, a high surface sensitivity appears close the waveguide corresponding to the thin bio-layer. Then, surface sensitivity will decrease with the increasing thickness of bio-layer and the next layer will eventually no longer change the propagation characteristics of the optical modes in the waveguide.

Due to the fact that surface sensitivity is not a constant, and that the processing of surface sensing is more complicated than bulk sensing, we still choose bulk sensing in the following verification part of the dual sensing of RI and pressure.

### 3.2. The Pressure Response of the MDR

When the microdisk device structure is under external pressure, the perimeter of the disk will be distorted, which changes *L* by *δL* due to the radial strain, and the effective RI (*n_eff_*) of the resonator will also vary due to the strain–optical (also called photo–elastic) effect. Both of the two changes will cause resonance wavelength shifts. Hence, they are both considered in our work. The shift of resonance wavelength is given by

(10) $$\frac{\delta \lambda }{\lambda }=\frac{\delta L}{L}+\frac{\delta n_{eff}}{n_{eff}}$$

where *δL* and *δn_eff_* stand for the variation in resonator perimeter and the effective refractive index, respectively.

**Figure 7:**
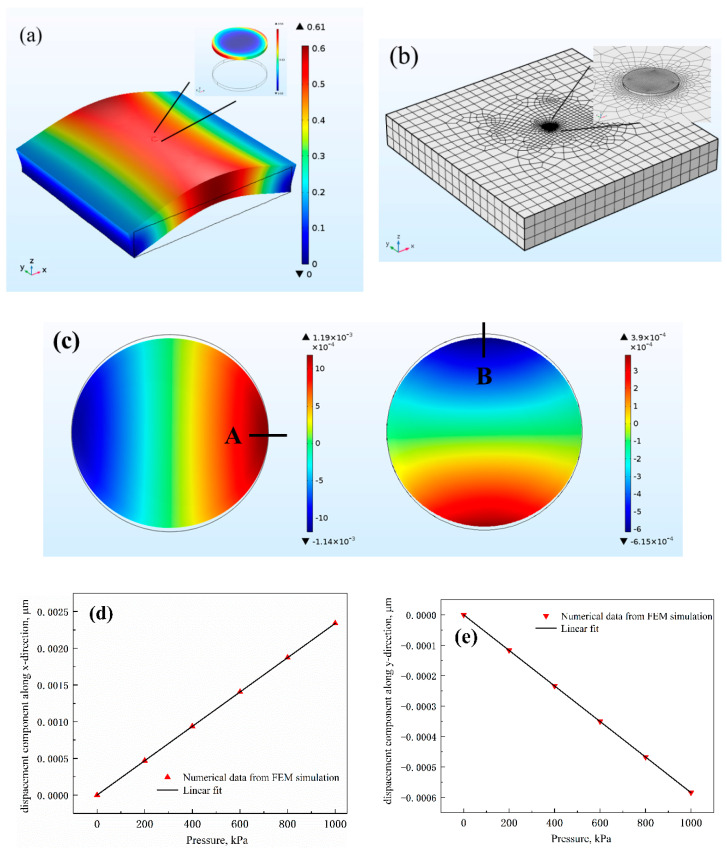
(**a**) The displacement of the substrate and the enlarged insert of MDR under a pressure of 600 kPa. (**b**) Mesh generation of the simulation model. (**c**) The displacement component distribution for the MDR in x and y direction. The x direction displacement component at point A, (**d**) and the y direction displacement component at point B (**e**) versus pressure.

First of all, we analyze the impact of dimensional changes on the resonance wavelength. When the substrate is bent, specific changes of deformation are obtained by performing the 3D finite-element method (FEM) mechanical simulation using the Solid Mechanics module by COMSOL Multiphysics. The substrate dimension is 18 × 18 µm and the free quad elements are selected for the mesh generation in the simulation model which is shown in Figure 7b. The basic material properties during the simulation are set as follows: for the silicon in the resonator device, Young’s modulus *E* = 130 GPa, Poisson’s ratio *υ* = 0.27 [39] and for SU-8 substrate, Young’s modulus *E* = 2 GPa, Poisson’s ratio *υ* = 0.22 [35]. The MDR occurs deformation in both the uniaxial strain direction and the direction perpendicular to the strain. The overall deformation of the whole structure when a uniform load of 600kPa is applied underneath the SU-8 layer is depicted in Figure 7a. The insert is the distinct deformation of MDR. From Figure 7c, it can be concluded that the disk is slightly stretched along the x-direction and compressed along the y-direction. Figure 7d,e show the displacement components along the x-axis at point A and y-axis at point B with different pressures exerted on the structure, representing a good linear relationship. Notably, the shape of the microdisk cavity changes slightly from a circle to an ellipse. Then the semi-major and -minor axes of the ellipse can be calculated according to the component of the displacement of the x- and y-axis, and it can be used as the initial size parameter of the microdisk resonator in the following 3D FDTD simulation.

Another effect factor for the change in the resonance wavelength is the effective RI. When applying a uniform load underneath the SU-8 layer, the total effective refractive index changes come from two aspects. One is caused by the dimension variation of the MDR, which has been studied in the analysis above, and the other is caused by the photo–elastic effect. The stress distribution within the MDR can influence the RI of the resonator to change, thus causing the variation of effective refractive index within it. The stress-induced variation in RI of resonator material can be expressed in the following mathematical model [39]

(11) $$\Delta n_{x}=n_{x}-n_{r}=-C_{1}\sigma_{x}-C_{2}\left(\sigma_{y}+\sigma_{z}\right)$$

(12) $$\Delta n_{y}=n_{y}-n_{r}=-C_{1}\sigma_{y}-C_{2}\left(\sigma_{x}+\sigma_{z}\right)$$

where *σ_x_*, *σ_y_* and *σ_z_* are the stress tensor components in the x-, y- and z-axes directions, respectively. The stress-optical constants *C*_1_ and *C*_2_ can set as −1.705 × 10^−11^ Pa^−1^ and 5.485 × 10^−12^ Pa^−1^ from the literature [38].

**Figure 8:**
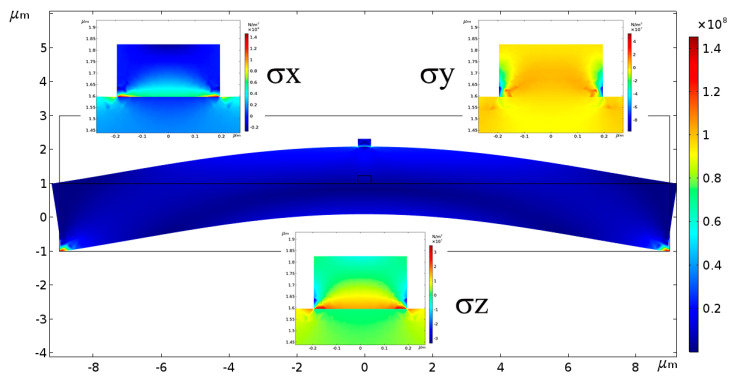
The diagram of the deformation of the microdisk waveguide under a pressure of 600 kPa from the COMSOL 2D FEM simulation. The inserts are the distribution of stress tensor components *σx*, *σy* and *σz*.

The stress tensor components can be numerically solved by 2D FEM mechanical simulation using the Solid Mechanics module by COMSOL Multiphysics. The dimension parameters of waveguide and substrate are respectively *w* × *h* = 390 × 230 nm and 18 × 2 μm. The prescribed displacement is set as the left and right vertices of the bottom of substrate are fixed. The Boundary Load is set as the uniform load is applied upward from the bottom of substrate. Figure 8 shows the distributions of *σ_x_*, *σ_y_* and *σ_z_* when a pressure of 600 kPa is applied underneath the substrate.

**Figure 9:**
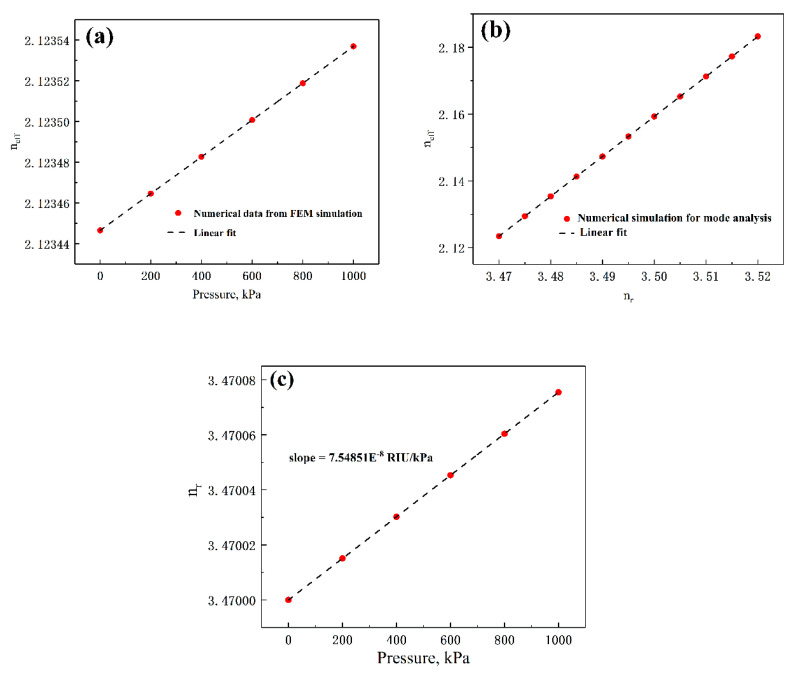
(**a**) The variation of *n_eff_* versus the applied pressure. (**b**) The relationship between the *n_eff_* and *n_r_* of MDR. (**c**) Increment of RI in Si resonator versus the applied pressure.

Combining the 2D FEM simulation of the mode analysis using the Wave Optics Module of COMSOL, the relationship between the *n_eff_* and the applied pressure is studied. Electromagnetic Waves Frequency Domain is chosen as the research COMSOL Physics. The effective refractive index is obtained using global equation of “ewfd.neff” and results are shown in Figure 9a. Then, the change of the *n_eff_* versus RI of the MDR (*n_r_*) can be obtained through the mode analysis of microdisk waveguide in Figure 9b. Through the mathematical calculation of the data in Figure 9a,b, it is found that the changes in the *n_r_* have a linear relationship with the applied pressure with a rate of 7.54851 × 10^−8^ RIU/kPa from Figure 9c.

**Figure 10:**
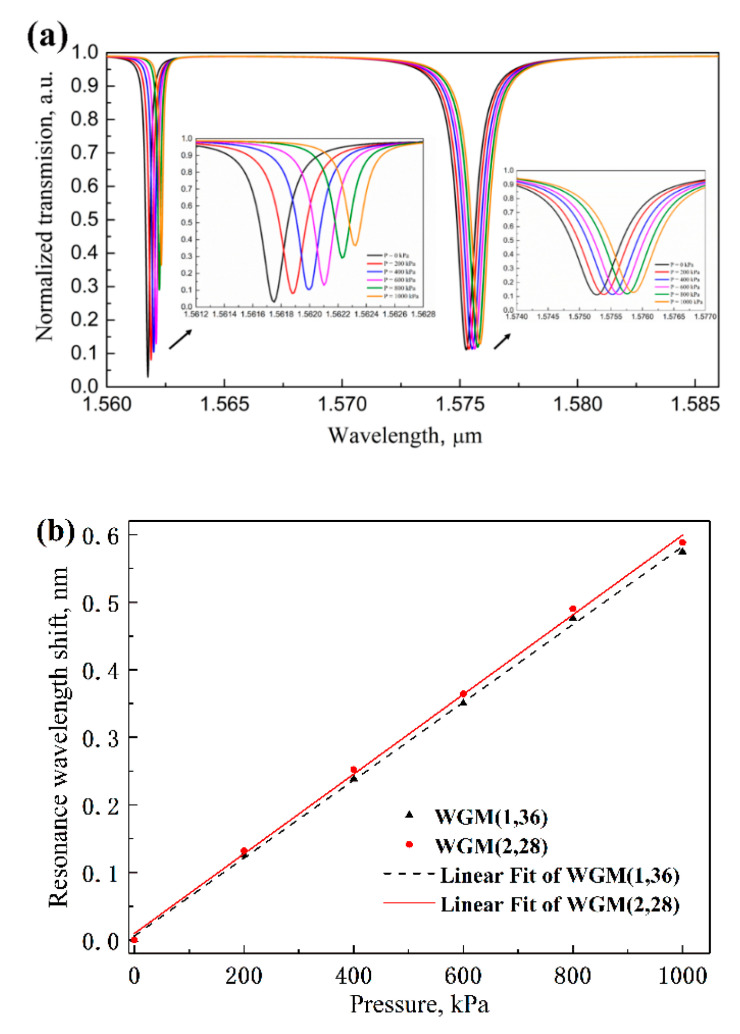
(**a**) The transmission spectra with different pressure ranging from 0 to 1000 kPa in a step of 200. (**b**) The linear fit of resonance wavelength shifts of the MDR versus the applied pressure.

Finally, according to the above analysis, the influence of pressure factor on the variations in the geometric dimension and the RI of the microdisk resonator are acquired by the 3D and 2D FEM simulations. Then these parameters of MDR are set in the simulation model for pressure sensing evaluation. The transmission spectrum with the applied uniform pressure ranging from 0 kPa to 1000 kPa in a step of 200 is shown in Figure 10a. Notably, the resonance wavelength shifts occur red-shift with the increase of the pressure for both two WGMs. From the linear fitting data shown in Figure 10b, the two pressure sensitivity is determined to be *S_P,WGM_*_(1,36)_ = 0.576 pm/kPa and *S_P,WGM_*_(2,28)_ = 0.589 pm/kPa. Based on Equation (7), when *F* is chosen with 1/100, the corresponding pressure detection limit is calculated to be around 4.524 and 22.283 kPa.

Therefore, we have obtained each sensitivity of the RI and pressure for the two WGMs to complete the sensing matrix *M_RI,P_*. Then, the RI and the pressure variations can be determined simultaneously by monitoring the resonance wavelength shifts of the WGM (1,36) and the WGM (2,28). Combined with Equation (10), the dual sensing results can be acquired by solving the inverse matrix equation:

(13) $$\left[\begin{array}{c} \delta n\\ \delta P \end{array}\right]={\left[\begin{array}{cc} 29.07 & 0.576\times 10^{-3}\\ 38.68 & 0.589\times 10^{-3} \end{array}\right]}^{-1}\times \left[\begin{array}{c} \delta \lambda_{WGM(1,36)}\\ \delta \lambda_{WGM(2,28)} \end{array}\right]$$

To test and prove the feasibility and the accuracy for the dual sensing performance based on the MDR, the set values of the changes in the ambient RI and the pressure (*δn_set_* and *P_set_*) are compared with the calculated values of that (*δn_cal_* and *P_cal_*) using Equation (13) through the obtained resonance wavelength shifts from the numerical simulations. For these four groups of measurements, the error between set values and calculated values is respectively 6.6 × 10^−4^, 8 × 10^−5^, 5.8 × 10^−4^, 1.18 × 10^−3^ for RI factor, and 12.072, 9.363, 21.938, 18.483 for factor pressure. In total, the average detection error for RI and pressure are within 6.25 × 10^−4^ RIU and 15.464 kPa, respectively, which agrees well with the detection limits we derived previously. All detailed results are shown in Table 1, which also exhibits a good agreement between the theoretical and the simulation solutions.

In Table 2, comparisons of sensing performance of optical resonators in dual-sensing for RI and temperature or RI and pressure are summarized. The proposed MDR has a higher *Q* except [36] and it possesses the best DL of RI compared to the other three resonators. As for pressure sensing, the *S_Pressure_* of MDR is smaller than that of SOMRR [33], but it has the comparable pressure DL due to the high quality factor. It can also be seen from the Table 2 that radius of the device is small, thus lead to a wider FSR which is suitable for large measurement range. Moreover, the small resonator size is beneficial for integration on chip.

## 4. Conclusions

In conclusion, a microdisk resonator (MDR) for measuring both the refractive index and the pressure simultaneously is proposed and verified by the 3D FDTD simulations. Due to the different energy distributions for WGM of the first two orders supported in the proposed MDR, which are denoted as WGM (1,36) and WGM (2,28), respectively, the responses to the RI and pressure changes show differences for two WGMs. For the WGM (1,36) and the WGM (2,28) with the corresponding high Q factor of 5990 and 1200, refractive index (RI) sensitivities of 29.07 and 38.68 nm/RIU, as well as pressure sensitivities of 0.576 and 0.589 pm/kPa are obtained according to the simulation results. Thus, we can detect the RI and pressure variations simultaneously by solving the second-order inverse sensitivity matrix. Therefore, the problem of inaccurate detection results due to the strain–optical coupling effect in flexible photonic biosensors can be effectively solved. This sensing scheme we proposed offers a new approach to overcome the difficulty of multi-sensing applications based on the flexible photonic device.

## Figures and Tables

**Table 1:** Dual sensing of RI and Pressure Based on the MDR

$\delta n_{set}$	$P_{\mathrm{set}}/\mathrm{kPa}$	$\delta \lambda_{WGM(1,36)}/\mathrm{nm}$	$\delta \lambda_{WGM(2,28)}/\mathrm{nm}$	$\delta n_{\mathrm{cal}}$	$P_{\mathrm{cal}}/\mathrm{kPa}$
0.026	200	0.845	1.091	0.02534	187.928
0.026	750	1.191	1.450	0.02592	759.363
0.048	200	1.481	1.939	0.04742	178.062
0.048	750	1.841	2.313	0.04682	768.483

**Table 2:** Comparisons of Sensing Performance of Dual-Sensing Optical Resonators.

Optical Resonator	Sensing Factor	Radius (μm)	Modes	Quality Factor	*S_RI_* (nm/RIU)	DL of RI (RIU)	*S_Pressure_* (pm/kPa)	DL of Pressure (kPa)	Ref
Microring	RI, Temperature	20	WGM (TE_0_) WGM (TM_0_)	5447 5462	104 319	1.2 × 10^−4^ 3.8 × 10^−4^	-	-	36
Microdisk	RI, Temperature	2	WGM (0,36) WGM (1,28)	~1500 ~200	45.8 72.9	8.59 × 10^−4^ 1.92 × 10^−3^	-	-	37
SOMRR	RI, Pressure	3	WGM (SM) WGM (AM)	738 395	77.07 69.54	2.72 × 10^−4^ 5.64 × 10^−4^	5.01 5.72	4.79 16.86	38
Microdisk	RI, Pressure	2	WGM (1,36) WGM (2,28)	5990 1200	29.07 38.68	8.96 × 10^−5^ 3.39 × 10^−4^	0.576 0.589	4.524 22.283	Our work
